# Different Circulating Trace Amine Profiles in *De Novo* and Treated Parkinson’s Disease Patients

**DOI:** 10.1038/s41598-019-42535-w

**Published:** 2019-04-16

**Authors:** Giovanni D’Andrea, Gilberto Pizzolato, Antonina Gucciardi, Matteo Stocchero, Giuseppe Giordano, Eugenio Baraldi, Alberta Leon

**Affiliations:** 1 Research and Innovation (R&I Genetics) s.r.l., Padova, Italy; 2 Department of Medical Sciences, Neurology Unit, University of Trieste, Trieste, Italy; 3 Mass Spectrometry and Metabolomic Laboratory, Women’s and Children’s Health Department, University of Padova, Padova, Italy; 4 Fondazione Istituto di Ricerca Pediatrica Cittàdella Speranza, Padova, Italy

## Abstract

Early diagnosis of Parkinson’s disease (PD) remains a challenge to date. New evidence highlights the potential clinical value of circulating trace amines (TAs) in early-stage PD and their involvement in disease progression. A new ultra performance chromatography mass spectrometry (UPLC-MS/MS) method was developed to quantify plasmatic TAs, and the catecholamines and indolamines pertaining to the same biochemical pathways. Three groups of subjects were recruited: 21 *de novo*, drug untreated, PD patients, 27 in treatment PD patients and 10 healthy subjects as controls. Multivariate and univariate data analyses were applied to reveal metabolic changes among the groups in attempt to discover new putative markers for early PD detection and disease progression. Different circulating levels of tyrosine (p = 0.002), tyramine (p < 0.001), synephrine (p = 0.015), norepinephrine (p = 0.012), metanephrine (p = 0.001), β-phenylethylamine (p = 0.001) and serotonin (p = 0.006) were found among the three groups. While tyramine behaves as a putative biomarker for early-stage PD (AUC = 0.90) tyramine, norepinephrine, and tyrosine appear to act as biomarkers of disease progression (AUC > 0.75). The findings of this pilot cross-sectional study suggest that biochemical anomalies of the aminergic and indolic neurotransmitters occur in PD patients. Compounds within the TAs family may constitute putative markers for early stage detection and progression of PD.

## Introduction

Parkinson’s disease (PD) is a progressive neurodegenerative disorder characterized by dopaminergic neuronal loss in the substantia nigra pars compacta resulting in a series of motor and non-motor disturbances. The motor deficits, which constitute the most important clinical sign for the diagnosis of PD, notoriously appear when approximately 60% of the dopaminergic neurons have been lost and the disease is already in the middle or late stage. The neuropathology of PD begins, in fact, years before the onset of the motor symptoms^1–3^. However, despite considerable progress in the field, clinically reliable biomarkers are still long sought for the detection of PD in the prodromal (clinical symptoms are present, but have not yet reached the criteria for diagnosis of PD) or preclinical (neurodegeneration has occurred but without clinical symptoms) phase, allowing for better prognosis and treatment of PD^4^.

In recent years significant research resources have been devoted to the search of clinical or neuroimaging biomarkers in PD, as well as possible other newer markers in biological samples^5^. Among the latter, metabolomic profiling has been employed as a tool to identify PD progression biomarkers, to discover novel biomarkers and to predict the development of PD^6^. Some clinical and experimental studies have reported changes in small molecules related to branched-chain amino acids and fatty acid metabolism, pointing to mitochondrial dysfunction in PD^7^.

**Figure 1:**
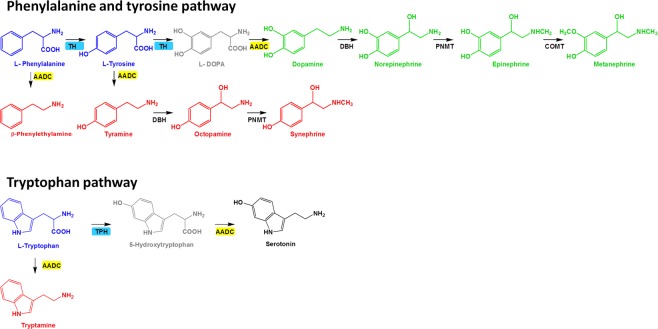
Chemical structure and biosynthesis pathways of trace amines (TAs). TAs, highlighted in red, include β-phenylethylamine (β-PEA), tyramine (TYRA), octopamine (OCT), synephrine (SYN), and tryptamine (TRY). They are synthesized from precursor aromatic amino acids (in blue), L-tyrosine (TYRO), L-phenylalanine (PHE) and L-tryptophan (TRP) by the aromatic-L-amino acid decarboxylase (AADC) together with catecholamines and metanephrine (MTN) (in green). Tyrosine hydroxylase (TH) converts PHE to TYRO and TYRO to L-DOPA, the precursor of dopamine (DA). TYRA and DA are metabolized by the dopamine β-hydroxylase (DBH) to OCT and norepinephrine (NE) respectively. The last two are converted by phenylethanolamine N-methyltransferase (PMNT) enzyme activity to SYN and epinephrine (E). TRP is the amino acid parent of serotonin (5-HT) and TRY, produced by the action of TRP hydroxylase (TPH) and decarboxylase (AADC) enzymes, respectively.

Recently, we provided evidence showing that a group of circulating amino acid-derived biogenic amines, defined as trace amines (TAs) or elusive amines, undergo early disease-related changes in PD^8^. TAs include β-phenylethylamine (β-PEA), tyramine (TYRA), octopamine (OCT), synephrine (SYN), and tryptamine (TRY)^9^. These compounds derive from tyrosine (TYRO), phenylalanine (PHE) or tryptophan (TRP) by the action of the carboxy-lyase L-aromatic amino acid decarboxylase (AADC) enzyme, also known as tyrosine, phenylalanine or tryptophan decarboxylase. In the same pathway catecholamines and indoleamines are also synthesized via the action of tyrosine (TH) and tryptophan (TPH) hydroxylases, as shown in Fig. 1. Although the role of TAs in the physiology of the nervous system is still largely elusive, their crosstalk with the catecholaminergic system together with the discovery of specific TAs receptors (TAARs) has called attention on their possible pathophysiological relevance in different CNS disorders. Studies conducted with knockout mice show that the TAAR1 receptors play a significant role in the control of movement as well as dopamine release from the nigrostriatal neurons^10,11^. In wild-type rodents, pharmacological blockade of TAAR1 increases the firing of dopaminergic neurons^12^. TAs are considered as full agonists of TAAR1^13^ and may play a role in the modulation of the central circuits controlling the dopaminergic system.

TAs are present in the central nervous system at very low concentrations in the order of 0.1–10 nmol/L^14^ and in peripheral tissues (0.1–100 ng/g tissue)^15^. Different methods have been described which allow for quantification of catecholamines and serotonin in biological samples^16^, including high pressure chromatographic (HPLC) and capillary electrophoresis coupled with fluorescence, ultraviolet, amperometric electrochemical detection (ECD) and mass spectrometry (MS). Only a few published papers describe the quantification of TAs in plasma. HPLC coupled with ECD has been used to measure TYRA, OCT, and SYN in human plasma and platelets^17^ while TRY has been measured by HPLC with fluorescence detection^18^. Although these techniques have high sensitivity and selectivity, they require time-consuming sample preparations and long chromatographic runs. Moreover, interfering peaks can be produced by endogenous substances in plasma with similar electrochemical or fluorescence properties and the evaluation of multiple compounds requires, at times, different assay methods.

To obtain a comprehensive evaluation of the levels of circulating TAs, catecholamines and related compounds in plasma of PD patients, we developed a new specific targeted metabolomics method based on ultra performance liquid chromatography-tandem mass spectrometry (UPLC-MS/MS). This approach has the advantage of a fast and high-efficiency separation system offered by the extremely small particle size of the UPLC column, along with the specificity and selectivity of MS/MS experiments with small amounts of samples employing labeled internal standards.

In this pilot cross-sectional study, we sought to confirm whether circulating TAs and the catecholamines and indolamines, involved in the same pathways, are altered in plasma of PD patients at different stages of the disease to preliminarily assess whether they could behave as metabolic markers in early and/or late-stage PD. Moreover, we also quantified the circulating levels of γ-aminobutyric acid (GABA), the main inhibitory neurotransmitter in the CNS, and adenosine (ADE), a compound potentially involved in neuroprotection^19^. Three groups of subjects were recruited: *de novo* patients (DN group) having disease duration less than 2 years and no history of present or past therapy with anti-parkinsonian drugs, in treatment patients (PD group) with a disease duration of less than 5 years under treatment with L-dopa or other dopaminergic drugs for more than 1 year, with a stable therapeutic response, and a control group consisting of age- and gender-matched healthy subjects with no clinical evidence of PD or other movement or neurodegenerative disorders (H group).

## Results

### Patients’ characteristics

A total of 48 patients were enrolled, of which 21 in the DN group (62% male and 38% female, mean age at enrollment 64 years, range 41–79 years) and 27 in the PD group (56% male and 44% female, mean age at enrollment 69 years, range 49–78 years). The mean duration of parkinsonian symptoms was 3.5 years (range 0.8–5.0) in the PD group and 10 months (range 6–24) in the DN group.

The control group consisted of 10 subjects (30% male and 70% female, mean age at enrollment 61 years, range 49–76 years).

No differences were found among DN, in treatment PD and H groups in age (Kruskal-Wallis test, p-value = 0.10) and sex (chi-square test, p-value = 0.24). No outliers were detected within the three groups on the basis of the preliminary PCA analysis of the measured metabolites and no significant effects of age and sex on the metabolite concentrations were found.

### Three group comparison

**Figure 2:**
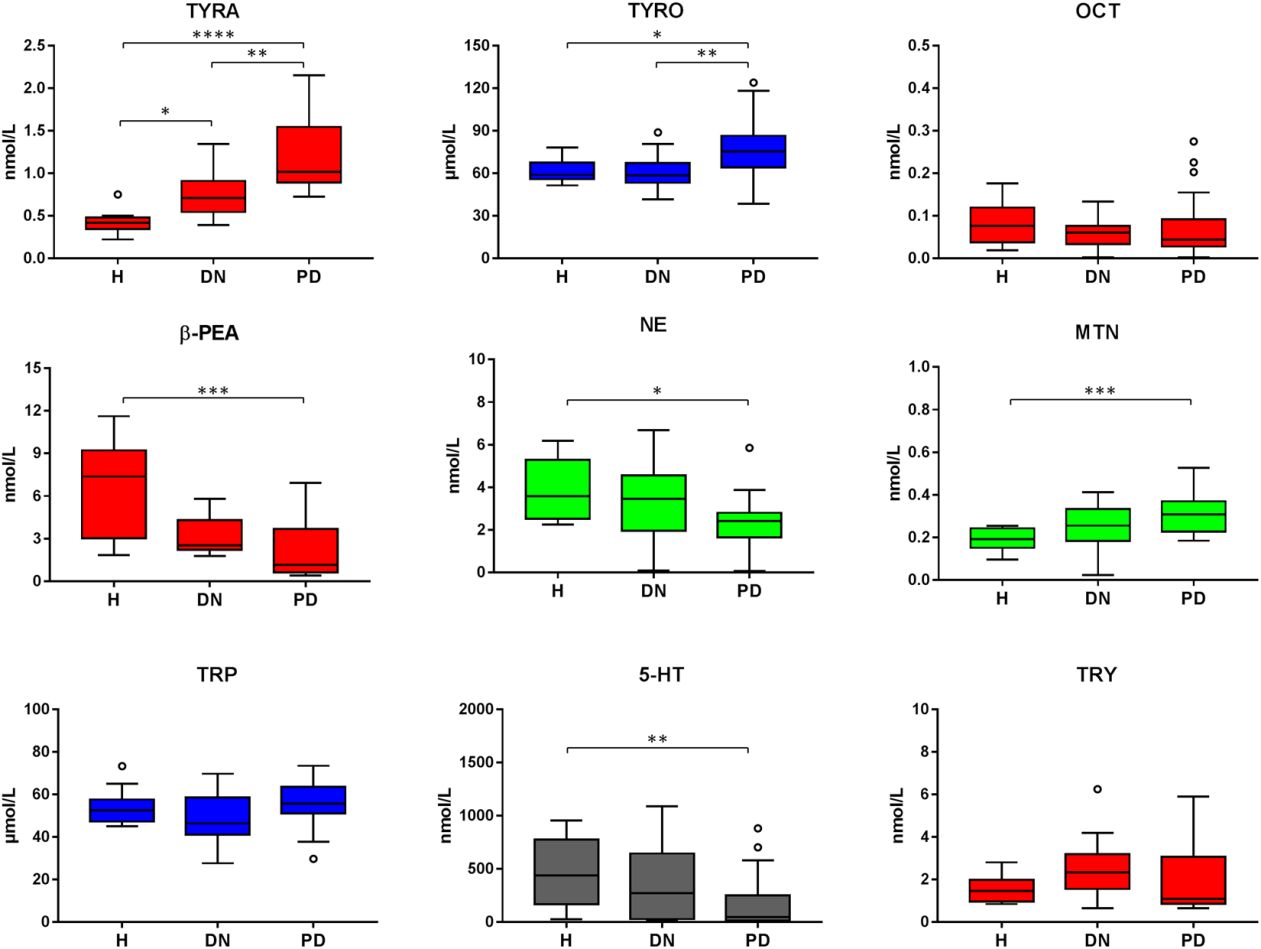
Comparison of plasma tyramine (TYRA), tyrosine (TYRO), octopamine (OCT), β-phenylethylamine (β-PEA), norepinephrine (NE), metanephrine (MTN), tryptophan (TRP), serotonin (5-HT) and tryptamine (TRY) in untreated *de novo* (DN) patients with treated PD patients (PD) and healthy controls (H). Horizontal lines represent median value, boxes represent interquartile range, and whiskers represent minimum-maximum values. Significant differences were obtained on the basis of the Kruskal-Wallis test followed by Dunn’s multiple comparisons test (****P < 0.0001; ***0.001 < P < 0.001; **0.001 < P < 0.01; *0.01 < P < 0.05). Detailed results and adjusted p-values are given in Table 1.

The plasmatic levels of the measured metabolites (TYRA, β-PEA, OCT, TRY, SYN, NE, 5-HT, E, MTN, ADE, GABA, TYRO, TRP) were compared among DN, PD and H groups by Kruskal-Wallis test followed by Dunn’s multiple comparisons test. We reported in Table 1 the results of the analysis. Significant differences were found in the medians of the plasmatic concentrations of some metabolites. Specifically, the PD group was characterized by higher levels of TYRA and TYRO and lower levels of NE than the *de novo* and healthy groups. β-PEA and 5-HT displayed higher levels in the H group than in the PD group, whereas MTN was higher in the PD group than in the healthy subjects. TYRA was significantly higher in the DN than in the healthy group. Box-plot distributions of the metabolites are reported in Fig. 2. Reference physiological values for plasmatic TAs are not available. Catecholamines, 5-HT, β-PEA, TRY and amino acids plasma concentrations measured in control subjects were in agreements with previously reported data^18,20–22^ whereas OCT levels were found lower than those previously reported^17^.

**Table 1:** Metabolite concentrations in plasma samples and three group comparison.

metabolite	H (n = 10)	DN (n = 21)	PD (n = 27)	Kruskal-Wallis p-value	H vs. DN p-value^a^	H vs. PD p-value^a^	DN vs. PD p-value^a^
TYRA	0.418 (0.344–0.481)	0.71 (0.547–0.910)	1.02 (0.895–1.546)	**<0.0001**	**0.02**	**<0.0001**	**0.002**
β-PEA	7.380 (3.029–9.222)	2.535 (2.214–4.322)	1.162 (0.613–3.687)	**0.001**	0.17	**0.0009**	0.18
OCT	0.077 (0.038–0.119)	0.061 (0.033–0.076)	0.044 (0.028–0.092)	0.57			
TRY	1.467 (0.971–1.991)	2.335 (1.567–3.204)	1.084 (0.868–3.068)	0.11			
SYN	0.016 (0.015–0.164)	0.051 (0.039–0.063)	0.194 (0.081–0.328)	**0.015**	1.00	0.12	0.19
NE	3.588 (2.521–5.294)	3.466 (1.966–4.555)	2.41 (1.645–2.801)	**0.012**	1.00	**0.03**	**0.05**
5-HT	441.5 (167.5–777.2)	272.6 (28.86–646.5)	49.3 (13.3–252.1)	**0.006**	0.73	**0.009**	0.10
E	0.289 (0.227–0.404)	0.279 (0.202–0.392)	0.267 (0.199–0.385)	0.69			
MTN	0.191 (0.151–0.243)	0.255 (0.183–0.334)	0.308 (0.228–0.370)	**0.001**	0.14	**0.0008**	0.19
ADE	218.7 (164.6–282.1)	209.8 (133.2–391.7)	187.00 (124.2–402.1)	0.88			
GABA	0.206 (0.181–0.234)	0.227 (0.196–0.259)	0.218 (0.182–0.261)	0.52			
TYRO	59.0 (55.8–67.7)	58.6 (53.4–67.4)	75.5 (64.2–86.5)	**0.002**	1.00	**0.04**	**0.003**
TRP	52.5 (47.3–57.7)	46.4 (41.1–58.7)	55.7 (51.2–63.7)	0.07			

Concentrations are reported as median and interquartile range (25–75%).

^a^Adjusted p-value obtained by Dunn’s multiple comparisons test.

**Figure 3:**
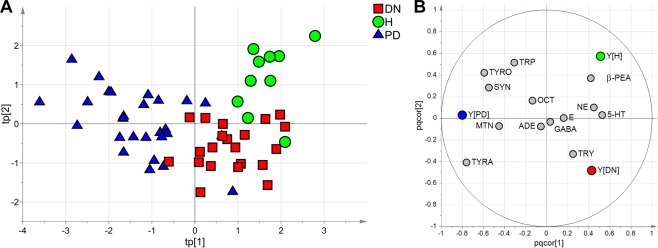
Multivariate data analysis. PLS-DA model after post-transformation^40^. In the score scatter plot (**A**), the symbols representing the samples of the investigated three groups belong to three different regions. The correlation loading plot (**B**) allows model interpretation in terms of metabolites characterizing each group.

Multivariate data analysis was applied to evaluate if the correlation structure of the measured variables could highlight differences in the three groups. A reliable PLS-DA model showing A = 3 components, R^2^ = 0.55 and Q^2^_CV 7-fold_ = 0.32 was obtained. Figure 3A shows the score scatter plot of the model. The plasma samples of the three groups lie in three different regions of the plot confirming that each group shows a different metabolic profile. Moreover, by analyzing the correlation loading plot of the model (Fig. 3B) and considering only the metabolites with significant Variable Influence on Projection, it was possible to qualitatively investigate the relationships between the groups and the concentration of the metabolites. Whereas the healthy group was characterized by lower levels of TYRA and higher levels of β-PEA than the patient groups, the treated PD group displayed lower levels of 5-HT and NE and higher levels of TYRA, TYRO, SYN, and MTN than the DN and healthy groups. Also, the DN group showed higher levels of TRY and lower levels of TRP than the healthy and the in treatment PD groups.

### *De novo* patient group (DN) versus healthy group (H)

Biochemical changes in *de novo* patients are of great interest because they are potentially useful for assessing the disease at an early stage. Therefore, we focused on the comparison between DN samples and those from healthy subjects.

The results of the ROC analysis are reported in Table 2.

**Table 2:** ROC analysis for the pair comparisons.

Variable	Type	AUC	CI AUC (95%)	Power	Specificity	Sensitivity
**DN vs**. **H**						
**TYRA**	**DN > H**	**0.902**	**0.784–1.000**	**0.998**	**0.90**	**0.86**
β-PEA	H > DN	0.719	0.484–0.953	0.528	0.60	0.90
OCT	H > DN	0.586	0.350–0.820	0.119	0.50	0.76
**TRY**	**DN > H**	**0.752**	**0.575–0.929**	**0.663**	**0.80**	**0.76**
SYN	H > DN	0.648	0.418–0.876	0.270	0.60	0.81
NE	H > DN	0.476	0.263–0.689	0.041	0.50	0.62
5-HT	H > DN	0.548	0.333–0.761	0.064	0.70	0.52
E	DN > H	0.519	0.306–0.731	0.037	0.50	0.57
MTN	DN > H	0.669	0.479–0.858	0.339	1.00	0.52
ADE	DN > H	0.486	0.281–0.690	0.034	0.80	0.38
GABA	DN > H	0.619	0.410–0.828	0.190	0.80	0.48
TYRO	H > DN	0.557	0.342–0.772	0.075	0.70	0.48
TRP	H > DN	0.690	0.501–0.879	0.417	0.90	0.57
**DN vs**. **PD**						
**TYRA**	**PD > DN**	**0.825**	**0.702–0.948**	**0.993**	**0.76**	**0.78**
β-PEA	DN > PD	0.566	0.391–0.740	0.124	0.71	0.63
OCT	PD > DN	0.516	0.346–0.685	0.039	0.57	0.67
TRY	DN > PD	0.644	0.480–0.806	0.417	0.76	0.63
**SYN**	**PD > DN**	**0.697**	**0.537–0.856**	**0.679**	**0.86**	**0.63**
**NE**	**DN > PD**	**0.757**	**0.612–0.900**	**0.902**	**0.67**	**0.89**
**5-HT**	**DN > PD**	**0.688**	**0.525–0.849**	**0.636**	**0.71**	**0.70**
E	DN > PD	0.594	0.429–0.759	0.207	0.71	0.56
MTN	PD > DN	0.641	0.474–0.807	0.404	0.48	0.81
ADE	PD > DN	0.504	0.336–0.672	0.028	0.57	0.48
GABA	DN > PD	0.563	0.397–0.728	0.115	0.57	0.59
**TYRO**	**PD > DN**	**0.775**	**0.640–0.910**	**0.943**	**0.71**	**0.78**
**TRP**	**PD > DN**	**0.674**	**0.513–0.833**	**0.567**	**0.67**	**0.78**

In the column Type the symbol “>” is used to indicate that the metabolite concentrations of the group on the left of the symbol is greater than that of the group on the right; AUC = area under the curve; CI AUC (95%) = confidence interval of the Area Under the ROC curve at the level of 95%; power = post hoc power of ROC analysis given α = 0.05; specificity and sensitivity correspond to the closest top left point of the ROC curve.

**Figure 4:**
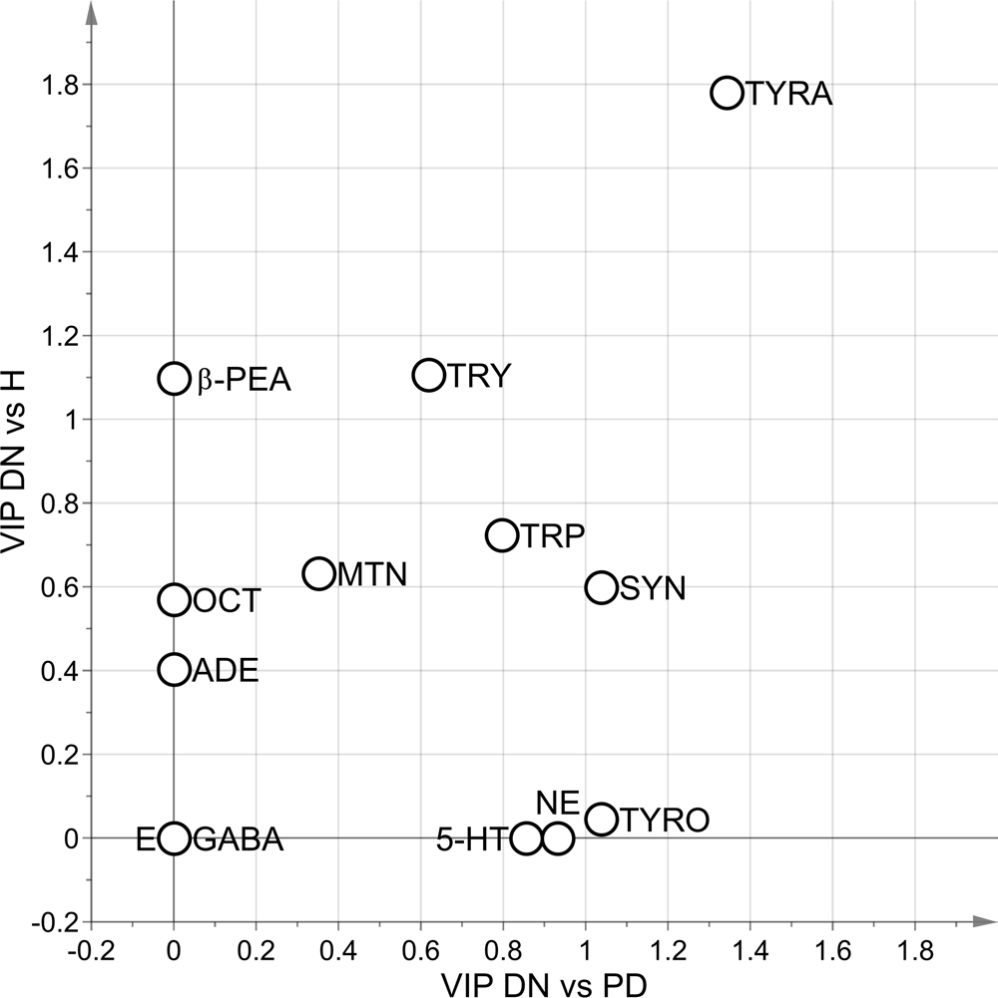
Median of the Variable Influence on Projection (VIP). VIP calculated for the PLS-DA VIP-based models generated during stability selection (500 random subsets) for the case DN vs. H (y-axis) and DN vs. H (x-axis); metabolites lying on one axis are not important in the discrimination of the groups considered in the other axis; the influence of the metabolite in the discrimination increases when the VIP increases.

The stability selection procedure was employed to delineate the role of the measured metabolites in characterizing the two groups under investigation. TYRA, β-PEA and TRY proved to be the most relevant variables for distinguishing the DN group from the H group. It is worth noting that OCT has an important role in the discrimination between the two groups due to the combined effects with the other TAs. In accordance with our previous report^8^, the mean level of OCT was greater in the H group than in the DN group. The median of the area under the ROC curve in prediction estimated by stability selection was 0.87 (5^th^ percentile equal to 0.65) raising the concrete possibility that the TAs, along with the catecholamines and indolamines considered, are promising markers for assessing the disease at an early stage. Figure 4 reports the results of the stability selection procedure.

**Figure 5:**
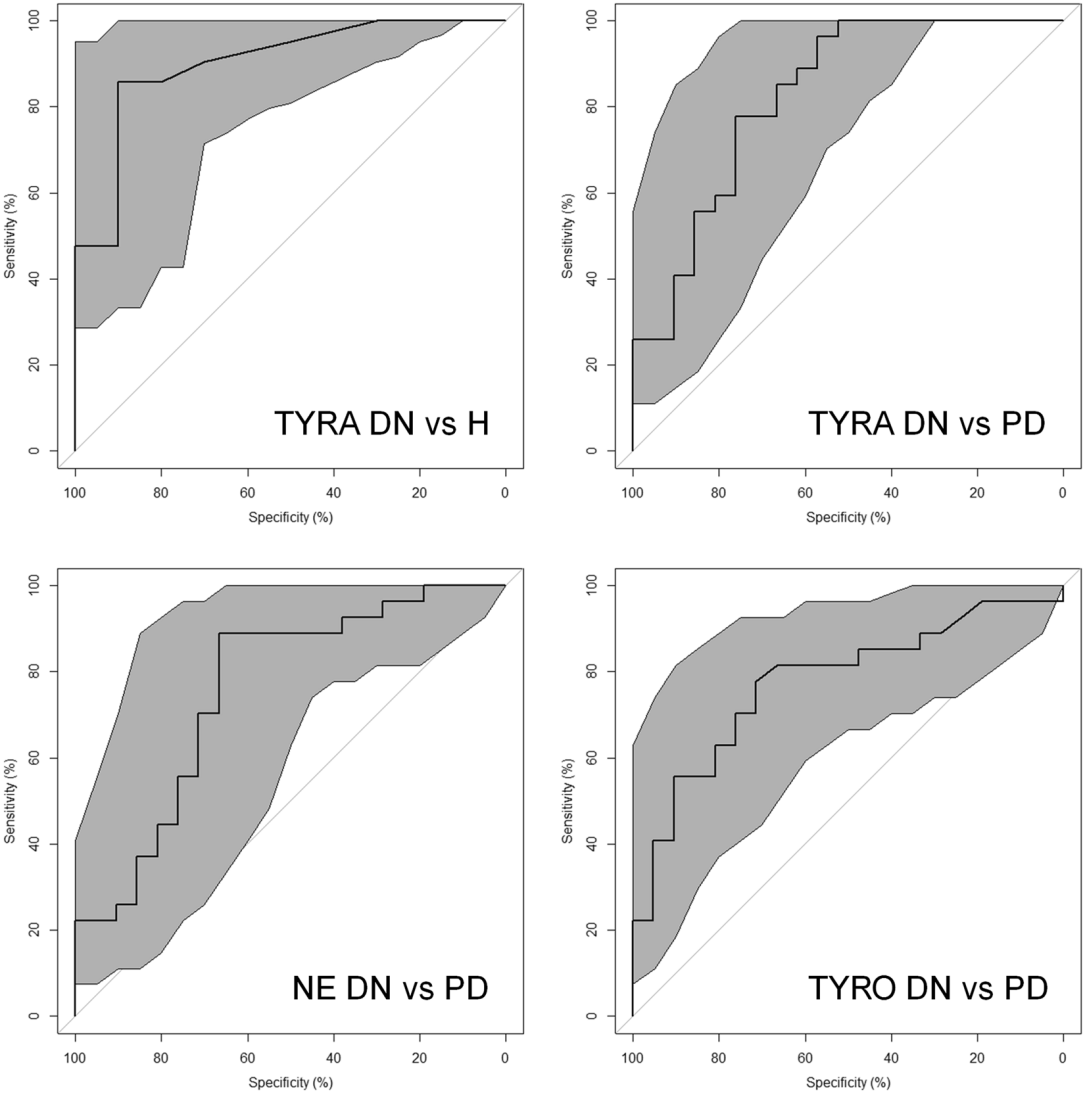
ROC curves. ROC curves of the metabolites showing power greater than 0.80; the grey region represents the confidence interval at the level of 95%.

Based on the combination of the results of ROC and multivariate data analysis, two metabolites, TYRA and TRY, were selected as putative markers. Table 3 reports their performance in discrimination determined on the basis of the ROC curve analysis. TYRA showed a power greater (>0.80), suggesting that this compound is a promising marker for assessing the disease at an early stage (in Fig. 5 we reported the ROC curve of TYRA).

**Table 3:** Parameters of the ROC curves of the relevant metabolites.

Variable	Type	AUC	CI AUC (95%)	Power	Specificity	Sensitivity
**TYRA**	**DN vs. H**	**0.902**	**0.784–1.000**	**0.998**	**0.90**	**0.86**
TRY	DN vs. H	0.752	0.575–0.929	0.663	0.80	0.76
**TYRA**	**DN vs. PD**	**0.825**	**0.702–0.948**	**0.993**	**0.76**	**0.78**
**TYRO**	**DN vs. PD**	**0.775**	**0.640–0.910**	**0.943**	**0.71**	**0.78**
**NE**	**DN vs. PD**	**0.757**	**0.612–0.900**	**0.902**	**0.67**	**0.89**
SYN	DN vs. PD	0.697	0.537–0.856	0.679	0.86	0.63
5-HT	DN vs. PD	0.688	0.525–0.849	0.636	0.71	0.70
TRP	DN vs. PD	0.674	0.513–0.833	0.567	0.67	0.78

AUC = area under the curve, CI AUC (95%) = confidence interval of the Area Under the ROC curve at the level of 95%; power = post hoc power of ROC analysis given α = 0.05; specificity and sensitivity correspond to the closest top left point of the ROC curve.

### *De novo* patient group (DN) versus in treatment patient group (PD)

To evaluate the metabolic differences related to PD disease progression, the metabolite levels in the in treatment PD group were compared to those in the DN patient group.

The results of the ROC analysis are reported in Table 2. The results of the stability selection procedure are reported in Fig. 4.

TYRA, SYN, and TYRO resulted to be the most relevant variables for distinguishing the DN group from the PD group. The median of the area under the ROC curves in prediction estimated by stability selection was 0.85 (5^th^ percentile equal to 0.62) demonstrating that the metabolic profile was able to explain the differences caused by the disease progression.

Combining the results of ROC and multivariate data analysis, six metabolites, TYRA, SYN, NE, 5-HT, TYRO, and TRP, were selected as putative markers. The performances in discrimination of the selected metabolites are reported in Table 3. TYRA, NE, and TYRO showed power greater than 0.80, raising the possibility that these compounds behave as promising markers for the progression of the disease (see Fig. 5 for the ROC curves for these metabolites).

## Discussion

This pilot cross-sectional study was designed to assess whether anomalies in the metabolism of trace amines and related organic compounds characterize different stages of PD and, as such, potentially constitute markers for early diagnosis and disease progression. By employing a new UPLC-MS/MS metabolomics method, we provide evidence that metabolic profiles resulting from the hydroxylation and decarboxylation of PHE, TYRO, and TRP are largely perturbed in PD patients.

These results raise the possibility that circulating levels of TYRA distinguish DN patients from healthy subjects whereas those of TYRA, NE and TYRO reflect the progression of the disease.

Trace amines are products resulting from the decarboxylation of the amino acids TYRO, PHE and TRP via the action of the AADC enzyme (also known as tyrosine, phenylalanine or tryptophan decarboxylase), whereas the classical neurotransmitters (DA, NE, and 5-HT) are hydroxylation products of the same amino acids via the enzymes TH or TPH (tyrosine or tryptophan hydroxylase), respectively (see Fig. 1)^13^. The function of AADC is regulated by the mitochondrial energy state and other factors related with monoaminergic function (i.e. the quantity of amines present in the competent synaptic clefts) and the number of surviving neurons containing AADC in the substantia nigra of PD patients^23^. By assessing the above enzyme products, we observed that increased circulating levels of TYRA and TYRO occur, together with lower levels of NE and 5-HT, in the treated PD group, while lower levels of β-PEA are found in both the DN and treated PD patient groups with respect to healthy controls.

One hypothesis to explain the high level of TYRA in DN patients, that is even more elevated in the treated PD patients, is that the activity of AADC enzyme is increased, an event which may reflects the reduced energy availability and mitochondrial dysfunction reported in PD^24–26^. Another possibility is that high TYRA levels may result from a slower rate of the catabolism by monoamine oxidase A (MAO-A)^27^. However, to date, reduced activity of this enzyme has not been shown in PD patients. Rather the low plasma levels of β-PEA, as previously reported, may be a consequence of increased catabolic monoamine oxidase B (MAO B) activity in PD patients^22,28^. On the other hand, the moderately low levels of OCT in both the PD patient groups may be a reflection of impaired function of DBH, involved in the transformation of TYRA to OCT^8,29^. The latter is supported by evidence reporting reduced activity of DBH in the brain of PD patients^30^.

Nonetheless, regardless of the metabolic pathways involved, our results show, to our knowledge for the first time, that circulating levels of TAs may constitute disease-related metabolic biomarkers in PD. Although we cannot exclude that pharmacological treatment with L-dopa, the substrate for DA production, may have interfered with TYRO metabolism in the patients with a long history of the disease, it is of noteworthy that elevation of TYRA and other amines also occur in the plasma of DN, patients free of treatment with L-Dopa or other antiparkinsonian drugs.

As expected, circulating NE levels were found to be reduced in the treated PD group, while no changes occurred in the DN group with respect to controls. This can be interpreted assuming that, in contrast to what happens for AADC in early PD, TH slowly decays during the course of the disease. However, it cannot be excluded that the decay of NE is a consequence of the high levels of MTN in treated patients. MTN is a product of activation of the COMT enzyme that transforms NE in MTN in the brain, medulla and sympathetic system^31^. MTN, as well as other amines, is an agonist of TAAR1 receptors^9^.

TPH transforms TRP into 5-HT, whereas TRY is the trace amine product of tryptophan decaboxylase^13^. Plasma levels of TRP tend to be lower in the DN than in the PD group, and higher in the treated PD group, whereas that of TRY are significantly higher in the DN and lower, together with that of 5-HT, in the treated PD group. These results support the hypothesis that analogously to the metabolic pathways involving tyrosine also that of tryptophan are perturbed. This raises the possibility that the decrease in the circulating levels of 5-HT found in patients with a long history of the disease may accompany the progression of the disease.

Should the changes in the circulating phenylalanine/tyrosine/tryptophan decarboxylase and tyrosine hydroxylase products mirror modifications in the CNS, the low levels of β-PEA, due possibly to an increase of MAO B activity, and the high levels of TYRA in the absence of changes in NE levels in the DN patients raises the possibility that different aminergic-containing neurons may regulate the neuro-vegetative function of the mammalian CNS^32^. Both β-PEA and TYRA play a role in the function of the sympathetic system that is probably the first target of CNS neuronal degeneration in PD^33^. Another important consideration is the possible impact of the anomalous high levels of TYRA and MTN, agonist on TAAR1 receptors^9^. These receptors modulate in an inhibitory manner the release of dopamine in the striatum and other dopaminergic systems^34,35^ an event ultimately resulting in reduced availability of the neurotransmitter in the synaptic clefts^36^.

Biomarkers of PD have long been dominated by measuring dopamine metabolites or alpha-synuclein in the cerebrospinal fluid. However, these markers do not allow early detection and monitoring of disease progression^7^. By employing pair comparisons for assessing biomarkers of early-stage disease and/or disease progression, we here also provide evidence that circulating levels of TYRA may be a promising, highly sensitive and specific (power >0.80), marker for early detection of PD, while those of TYRA, NE, and TYRO may cumulatively constitute putative markers of disease progression (power >0.80). Although further confirmatory studies are needed, the circulating levels of these latter compounds were found to have robust statistical power in discriminating between early and late-stage PD.

This study was a pilot study and has several limitations. Although DN patients were treatment-free, patients with late-stage PD were in treatment with L-dopa or other dopaminergic drugs. Because treatment could not be halted, we do not know to what degree this may have influenced the results. Moreover, our findings should be replicated and confirmed in an independent larger study of PD patients at different stages of the disease to confirm the results and lead to clinically useful information, also in view of the fact that we did not include a blind test set but applied only internal validation procedures.

In sum, our results show that PD is characterized by profound changes in the levels of the aminergic and indolic neurotransmitters. These changes, when assessed in the circulation, have the potential to provide for disease biomarkers, either alone or in combination with other markers. Also, should these changes mirror ongoing changes in the hypothalamus and other dopaminergic centers within the CNS, it is possible that pharmacological agents modulating TAAR receptors may represent new avenues in the prevention and/or treatment of PD.

## Methods

### Standard protocol approvals, registrations, and patient consents

The study was approved by the ethics committee of University Hospital “Ospedali Riuniti” of Trieste, Italy (protocol number 209/2012) and was performed in accordance with the Helsinki declaration. All participants were informed in writing and orally about the project and gave written consent prior to participation.

### Participants

The neurologists at the Movement Disorders Center of Neurology Clinic at University of Trieste, Italy, examined a consecutive series of patients with symptoms suggestive or already with a clinical diagnosis of PD according to the United Kingdom Parkinson’s Disease Society Brain (UK PDS) Bank Diagnostic Criteria for Parkinson’s Disease. Study inclusion was from October 1 2012 through December 30 2013. A total of 48 patients were enrolled in this pilot cross-sectional study. The recruited subjects were classified into two groups: *de novo* patients (DN group, n = 21) having disease duration less than 2 years and no history of present or past therapy with anti-parkinsonian drugs, and in treatment patients (PD group, n = 27) with a disease duration of fewer than 5 years being under treatment with L-dopa or other dopaminergic drugs for more than 1 year, with a stable therapeutic response (no wearing off or on-off phenomena or other fluctuations in the therapeutic response).

Exclusion criteria for patients were: vascular basal ganglia lesions on CT/MRI evaluation, symptoms suggestive of a diagnosis of Parkinson’s Plus Syndrome (e.g. ataxia, ocular movements disorders, relevant dysautonomia), clinically evident cognitive impairment (MMSE score ≤ 24), depressive disorder, intake of neuroleptics or other drugs, other than anti-Parkinson drugs in the PD group, interfering with the dopaminergic neurotransmission in the previous six months.

Moreover, a control group consisted of 10 age- and gender-matched healthy subjects with no clinical evidence of PD or other movement or neurodegenerative disorders (H group) was enrolled among individuals traveling with the patients.

### Samples collection

Blood sampling (20 ml) were collected from each subject in the supine position in a quiet room, and at the same time of day (between 9.00 and 11.00 in the morning) following a fasting period. The blood was collected from the basilic vein and immediately centrifuged. The plasma was transferred in 1 ml tube, serially numbered, assigned an anonymous code corresponding to the patient’s identity and promptly frozen at −80 °C. Collected samples were sent in frozen bags to the Laboratory of Mass Spectrometry and Metabolomics, at Women’s and Children’s Health Department, University of Padova, Italy.

### Chemicals and reagents

L-norepinephrine hydrochloride, (±)−dopamine hydrochloride, serotonin hydrochloride, epinephrine hydrochloride, tryptamine, (±)−octopamine hydrochloride, (±)−synephrine, tyramine hydrochloride, β-phenethylamine, D,L-metanephrine hydrochloride, adenine riboside, 4-aminobutyric acid, L-tyrosine, L-tryptophan, L-tryptophan-d5 (TRP-D5) and 4-aminobutyric acid-2,2,3,3,4,4-d6 (GABA-D6) were purchased from Sigma-Aldrich Corporation (Milan, Italy). The deuterated internal standards, (±)−norepinephrine-2,5,6,α,β,β-d_6_ HCl (NE-D6), 2-(3,4-dihydroxyphenyl)ethyl-1,1,2,2-d_4_-amine (DA-D4), serotonin-α,α,β,β-d_4_ creatinine sulfate complex (5HT-D4), (±)−epinephrine-2,5,6,α,β,β-d_6_ (E-D6), tryptamine-α,α,β,β-d_4_ HCl (TRY-D4), (±)−p-octopamine-α,β,β-d_3_ HCl (OCT-D3), 2-(4-hydroxyphenyl)ethyl-1,1,2,2-d_4_-amine HCl (TYRA-D4), and 2-phenyl-d_5_-ethylamine (β-PEA-D5) were obtained from CDN Isotopes (Pointe-Claire, Quebec, Canada). The L-tyrosine-ring-d4 (TYRO-D4) was from Cambridge Isotope Laboratories, Inc. (Tewksbury, MA, USA). The purity of all analytes and labeled internal standards was ≥98%.

Water was purified with a Milli-Q Elix purification system (Millipore, Bedford, MA, USA). High-purity MS-grade solvents (formic acid, methanol, and acetonitrile) were obtained from Fluka (Milan, Italy) and used without further purification.

### Preparation of standard solutions and calibration curves

Individual stock solutions of NE and E were prepared in methanol with 5% formic acid; TYRO and 5-HT have been dissolved in 0.1 M HCl in water; β-PEA, TYRA, OCT, SYN, MTN, TRY were in methanol; ADE, TRP, and GABA stock were prepared in water. A series of solution mixtures of desired concentrations were prepared by suitable dilutions of the stock solutions in 0.1% formic acid in water. All the stocks were stored at −20 °C.

Stock solutions of labeled metabolites were prepared as the unlabeled and diluted as required, with water 0.1% FA, to obtain a concentration of 0.05–0.1 µM for neurotransmitters and 100–200 µM for amino acids, and used as internal standard (IS).

Calibration curves of the analytes were prepared by spiking pooled plasma, obtained from volunteers, with the diluted mixed standard solutions and IS, to the concentration ranging from 0.1 to 4000 nmol/L for neurotransmitters and from 1 to 200 µmol/L for amino acids.

### Sample preparation

For the neurotransmitters analysis, a solid phase extraction (SPE) protocol on WCX cartridges 25 mg/ 1 mL (AN739) provided by Biotage (Uppsala, Sweden) to extract catecholamines from plasma was optimized to improve sensitivity and reduce limits of quantification. Briefly, WCX SPE cartridges were conditioned with 1 mL methanol, followed by equilibration with 1 mL 50 mmol/L ammonium acetate at pH 6. The samples, the calibration curve, and the quality controls were prepared by adding 100 μL of labeled IS solution and 600 μL of 50 mM ammonium acetate in water at pH 6 to 600 μL of plasma, and applied to the cartridges after mix. Each cartridge was twice washed with 900 µL of methanol 10% in water. After washing, the cartridges were eluted with 900 μL and 600 µL of 5% formic acid in methanol. The eluent was evaporated to dryness under nitrogen flow at 40 °C. Samples were reconstituted with 100 μL of 0.1% formic acid in water, and 5 μL were injected for UPLC-MS/MS analysis.

For the amino acids analysis, 10 µL of plasma, calibrator and quality control sample were spiked with 100 µL of a methanolic solution of labeled IS. After protein precipitation, the supernatant was evaporated and was subsequently reconstituted in 100 µL of 0.1% formic acid in water, and 5 μL were injected for UPLC-MS/MS analysis.

### UPLC-MS/MS method

The method was developed on a Xevo TQ-S MS/MS System coupled to an Acquity UPLC (Waters Milford, MA, USA).

Mobile phase A was water with 0.1% formic acid, and mobile phase B was methanol: acetonitrile 90:10 with 0.1% formic acid. Samples were injected onto a UPLC HSS PFP column (100 Ǻ, 1.8 µm, 2.1 mm × 100 mm) at a flow rate of 0.2 mL/min. Mobile phase A and mobile phase B were operated with gradient elution as follows:  99.5% A at 0–2 min; 99.5% to 90% A at 2–4.5 min; 90% to 75% A at 4.5–7 min; 75% to 50% A at 7–8.5 min; 50% to 30% A at 8.5–9.5 min, hold until 10 min; 30% to 10% A at 10–11 min, 10% to 99.5% A at 11–15 min. The total run time was 15 min. The acquisition was set in positive electrospray ionization mode with multiple reaction monitoring (MRM). The acquisition parameters were set individually for each analyte and are summarized in Table S1 of Supplementary Material. Instrument control, acquisition and the analysis of data were provided by MassLynx (version 4.1, Waters).

### Performance characteristics of the analytical method

The assay was found to be linear for all analytes under investigation over the concentration range with R2 > 0.990. Intra-day and inter-day precision and accuracy of the analytes were well within acceptance criteria (±15%). The matrix effect was evaluated. The ion chromatogram profile of plasma samples show an area of ion suppression from 1.2–1.8 minutes and the peak areas of GABA and related labeled IS were reduced by the ion suppression effect. No significant matrix effects for other analytes and IS were observed.

The limit of quantification (LOQ) was proved to be the lowest concentration on the calibration curve with a signal-to-noise ratio greater than ten. LOQ for amino acids was largely under plasmatic concentration levels (0.1 µmol/L for TYRO, TRP, GABA, 50 nmol/L for adenosine). LOQ established for TAs and catecholamines was sufficient to perform the quantification in plasma samples (0.20 nmol/L for NE, 0.13 nmol/L for E and TYRA, 0.15 nmol/L for DA, 0.05 nmol/L for 5-HT, β-PEA, and SYN, 0.10 nmol/L for OCT, 0.26 nmol/L for TRY). The concentration of OCT and SYN resulted to be lower than the LOQ for most of the collected sample and, then, their concentration should be considered as semi-quantitative.

### Statistical methods and data analysis

Our study was a pilot cross-sectional study. No a priori information was available to perform a power analysis. In this respect, data analysis should be interpreted as exploratory. Again, model validation was performed by internal validation procedures as described in the following.

Demographic data were investigated by chi-square test and Kruskal-Wallis test.

Statistical data analysis aimed to characterize the groups of subjects under investigation was performed by univariate and multivariate data analysis.

As the first step of data analysis, Principal Component Analysis (PCA) was applied to every single group to highlight the presence of outliers whereas the effects of age, sex, and group on the concentration of the measured metabolites were investigated by Multiple Linear Regression (MLR) with Box-Cox transformation. PCA is a multivariate unsupervised technique that produces new data representations where the so-called principal components, obtained linearly combining the measured variables, are used to summarize the information contained in the data^37^. Trends in the data, clusters of observations and outliers can be detected exploring the latent space and the residuals of the PCA model. On the other hand, MLR is a multivariate regression technique that allows the investigation of the relationships between factors (age, sex, and group) and response (metabolite concentration) taking into account the effect of all the factors at the same time. The Box-Cox transformation has been applied to have normally distributed residuals in order to perform an accurate statistical inference. MLR cannot be applied in the case of strongly correlated factors because the variance of the regression coefficients becomes very large making it difficult to obtain precise estimates of the separate effects of the factors, and large errors in prediction are obtained. Under this condition, Projection to Latent Structures (PLS)-based methods should be preferred.

Thus, we compared the three groups together. Kruskal-Wallis test followed by Dunn’s multiple comparisons test was performed and a global Projection to Latent Structures-Discriminant Analysis (PLS-DA) model was built. PLS-DA is a multivariate classification technique that is usually applied when the factors (i.e. the measured metabolites) are strongly correlated^38^. The measured variables are linearly combined to obtain a new set of variables, called latent variables, which are able to discriminate the observations. N-fold cross-validation (CV) is applied to estimate the power in prediction of the PLS-DA model and determine the number of latent variables to be calculated. The parameter Q^2^_CV N-fold_, that has the same form of the goodness-of-fit R^2^ but uses the predicted responses during CV instead of the calculated responses, is used to describe the performance of the model. For the PLS-DA model, the number of components was determined on the basis of the first maximum of Q^2^_CV 7-fold_. N-fold cross-validation (N = 6, 7, 9) and permutation test on the class response were applied to avoid overfitting according to good practice for model building.

Finally, we considered the *de novo* group as a control group and performed pair comparisons with the healthy group and the group of treated patients in order to discover putative markers for the disease and for its progression, respectively. Receiver Operating Characteristic (ROC) analysis and PLS-DA with stability selection based on Monte-Carlo sampling^39^ were applied to investigate the differences between the groups under investigation and discover putative markers. The central idea of stability selection is that real data variations should be present consistently, and therefore should be found even under perturbation of the data by sub-sampling. During stability selection, 500 random subsamples of the collected samples were extracted by Monte-Carlo sampling (with a prior probability of 0.70), and then PLS-DA VIP-based was applied to each subsample, obtaining a set of 500 discriminant models. The Variable Influence on Projection (VIP) score has been introduced to measure the importance of a variable in the PLS-DA model. In PLS-DA VIP-based a variable selection procedure based on VIP is implemented. Specifically, all the variables showing VIP under a given threshold are excluded during model building. The threshold to use has been defined on the basis of the best Q^2^_CV 7-fold_. The predictive performance of each model was estimated by means of ROC analysis of the outcomes of the predictions of which samples would be excluded during sub-sampling. Within the set of PLS-DA VIP-based models obtained by stability selection, the most frequently selected variables were identified as relevant variables. Significance levels were set to α = 0.05.

Univariate data analysis was performed by GraphPad Prism 7 software (GraphPad Software Inc., La Jolla, CA, USA) or SPSS Statistics 23.0 (SPSS Inc., Chicago, IL, USA). PCA and PLS-DA models were built by SIMCA 15 (Sartorius Stedim Data Analytics AB, Umeå, Sweden) whereas MLR, stability selection and ROC curve analysis were performed by R 3.3.2 platform (R Foundation for Statistical Computing, Vienna, Austria).

## Supplementary information

Supplementary material

## Data Availability

Anonymized data will be shared by request from any qualified investigator.
