# Supplementary material for: Different Circulating Trace Amine Profiles in De Novo and Treated Parkinson’s Disease Patients
Source: Sci Rep. 2019 Apr 16;9:6151. doi: 10.1038/s41598-019-42535-w (PMC6467876; doi:10.1038/s41598-019-42535-w)
Supplement: Supplementary file 1 — Supplementary material [file 41598_2019_42535_MOESM1_ESM.pdf]

SUPPLEMENTARY MATERIAL

Different circulating trace amine profiles in *de novo* and treated Parkinson's disease patients.

Giovanni D'Andrea, Gilberto Pizzolato (deceased), Antonina Gucciardi, Matteo Stocchero, Giuseppe Giordano, Eugenio Baraldi, and Alberta Leon.

Table S1: MRM transitions and chromatographic retention times for compounds and relative labeled internal standard measured in the study

Analytes	Precursor ion (*m/z*)	Product ion (*m/z*)	Retention time (min)	Dwell time (s)	Cone (V)	Collition energy (eV)
GABA	104.08	87.09	1.84	0.025	20	10
GABA-D6	110.20	93.11	1.84	0.025	20	10
Norepinephrine	170.20	107.06	2.11	0.025	10	16
Norepinephrine-D6	176.20	111.11	2.11	0.025	10	16
Octopamine	136.08	91.23	2.52	0.025	20	15
Octopamine-D3	139.03	121.07	2.52	0.025	20	10
Epinephrine	184.11	166.09	2.99	0.025	10	20
Epinephrine-D6	190.25	172.15	2.99	0.025	10	20
Tyrosine	182.20	136.10	3.36	0.025	10	12
Tyrosine-D4	186.10	140.20	3.36	0.025	10	12
Synephrine	150.10	91.20	4.09	0.039	20	20
Tyramine	138.08	121.08	4.95	0.034	20	10
Tyramine-D4	142.20	125.2	4.95	0.025	20	10
Adenosine	268.08	136.06	5.83	0.025	5	20
Metanephrine	180.10	148.10	5.94	0.025	20	20
Metanephrine-D3	183.10	151.10	5.94	0.025	20	20
Serotonin	177.10	160.10	7.34	0.025	30	30
Serotonin-D4	181.14	164.13	7.34	0.025	30	30
Phenylethylamine	122.03	105.12	7.90	0.025	10	10
Phenylethylamine-D5	127.01	110.01	7.90	0.025	10	10
Tryptophan	205.03	146.05	8.94	0.025	10	16
Tryptophan-D6	210.03	151.05	8.94	0.025	10	16
Tryptamine	161.10	144.10	9.74	0.025	10	10
Tryptamine-D4	165.20	148.13	9.74	0.025	10	10
